# The Benefits of the Post-Transplant Cyclophosphamide in Both Haploidentical and Mismatched Unrelated Donor Setting in Allogeneic Stem Cells Transplantation

**DOI:** 10.3390/ijms24065764

**Published:** 2023-03-17

**Authors:** Jarosław Dybko, Małgorzata Sobczyk-Kruszelnicka, Sebastian Makuch, Siddarth Agrawal, Krzysztof Dudek, Sebatian Giebel, Lidia Gil

**Affiliations:** 1 Department of Hematology and Cellular Transplantation, Lower Silesian Oncology Center, 53-413 Wroclaw, Poland; 2 Department of Bone Marrow Transplantation and Oncohematology, Maria Sklodowska-Curie National Research Institute of Oncology, Gliwice Branch, 44-102 Gliwice, Poland; 3 Department of Clinical and Experimental Pathology, Wroclaw Medical University, 50-368 Wrocław, Poland; 4 Department and Clinic of Internal Medicine, Occupational Diseases, Hypertension and Clinical Oncology, Wroclaw Medical University, 50-556 Wrocław, Poland; 5 Statistical Analysis Center, Wroclaw Medical University, 50-368 Wroclaw, Poland; 6 Department of Hematology and Bone Marrow Transplantation, Poznan University of Medical Sciences, 60-569 Poznań, Poland

**Keywords:** MMUD-HSCT, PTCy, GvHD, prophylaxis, transplantation, survival

## Abstract

Allogeneic hematopoietic cell transplantation (alloHSCT) is a standard therapeutic approach for acute leukemias and many other hematologic malignancies. The proper choice of immunosuppressants applicable to different types of transplantations still requires strict and careful consideration, and data in this regard are divergent. For this reason, in this single-centered, retrospective study, we aimed to compare the outcome of 145 patients who received post-transplant cyclophosphamide (PTCy) for MMUD and haplo-HSCT or GvHD prophylaxis for MMUD-HSCT alone. We attempted to verify if PTCy is an optimal strategy in MMUD setting. Ninety-three recipients (93/145; 64.1%) underwent haplo-HSCT while 52 (52/145; 35.9%) underwent MMUD-HSCT. There were 110 patients who received PTCy (93 in haplo and 17 in MMUD group) and 35 patients received conventional GvHD prophylaxis based on antithymocyte globulin (ATG), cyclosporine (CsA), and methotrexate (Mtx) in the MMUD group only. Our study revealed that patients receiving post-transplant cyclophosphamide (PTCy) show decreased acute GvHD rates and CMV reactivation as well as a statistically lower number of CMV copies before and after antiviral treatment compared to the CsA + Mtx + ATG group. Taking into account chronic GvHD, the main predictors are donor age, ≥40 years, and haplo-HSCT administration. Furthermore, the survival rate of patients following MMUD-HSCT and receiving PTCy with tacrolimus and mycophenolate mofetil was more than eight times greater in comparison to patients receiving CsA + Mtx + ATG (OR = 8.31, *p* = 0.003). These data taken together suggest that the use of PTCy displays more benefits in terms of survival rate compared to ATG regardless of the type of transplantation performed. Nevertheless, more studies with a larger sample size are required to confirm the conflicting results in the literature studies.

## 1. Introduction

Allogeneic hematopoietic cell transplantation (allo-HSCT) is a gold-standard curative therapy for acute leukemias and many other hematologic malignancies. The best possible outcomes are observed in the setting of well-matched donors [1]. However, despite the increasing number of volunteers registering as donors, transplant access remains a substantial public health problem, especially for some ethnic and racial minorities [2,3]. Most centers would rather recommend matched unrelated donors (MUD) than haplo if available in a timely manner. For instance, a large CIBMTR/EBMT study for lymphoma demonstrated better outcomes for MUD than for haplo [4]. On the other hand, a Korean study for acute leukemia showed no inferiority of haplo setting [5]. The problem starts when no sibling nor MUD donor can be found. Alternative options rely on transplantation with less-than-fully-matched donors, including haploidentical (haplo) donors, mismatched unrelated donors (MMUD), or unrelated cord blood units [2,6,7]. Haploidentical HSCT was proven to be effective with acceptable toxicity and mortality with the use of post-HSCT high-dose cyclophosphamide [8,9], although large studies favor matched unrelated over a haploidentical donor in spite of using PTCy [4,10]. The data with mismatched unrelated versus haploidentical are ambiguous and depend on HLA (human leukocyte antigen) matching and diagnosis. Nevertheless, the increasing experience with these strategies has led to the enhanced risk of graft-versus-host disease (GvHD) prophylaxis, higher nonrelapse mortality (NRM), reduced relapse-free survival (RFS), and overall adverse survival (OS) when compared with matched donor transplants [1,2,7]. Furthermore, it is still not clear which of these options are superior to the other ones [11].

An effective strategy to overcome HLA disparity, improve GvHD rates, and induce immune tolerance is to intensify immunosuppression using in vivo T-cell-depleting agents. The current literature is based on a combination of various agents for GvHD prophylaxis. Among the most effective compounds in haplo-HSCT is cyclophosphamide (PTCy) in combination with tacrolimus (TAK), and mycophenolate mofetil (MMF) [12,13,14,15]. Accumulating data from several studies has proven their application in reliable engraftment, and a lower incidence of GvHD in matched [16,17] and mismatched donor settings such as haplo-HSCT [18]. Due to these promising results in haplo-HSCT, studies have subsequently been extended to other donor types, including MMUD-HSCT, where conventional GvHD prophylaxis often included anti-thymocyte globulin (ATG), in association with other immunosuppressive agents [19,20]. A few single-center studies have recently compared PTCy and ATG for GvHD prevention in HLA-MMUD transplants in a variety of diseases. 

As Battipaglia et al. [7] suggested in their work that a large analysis of MMUD versus haplo-HSCT is warranted. We performed a study aiming to compare the outcomes of 145 patients who received post-transplant cyclophosphamide (PTCy) for MMUD and haplo-HSCT or “conventional” GvHD prophylaxis for MMUD-HSCT alone. We tried to find out if PTCy is an optimal strategy in an MMUD setting. 

## 2. Results

**Figure 1:**
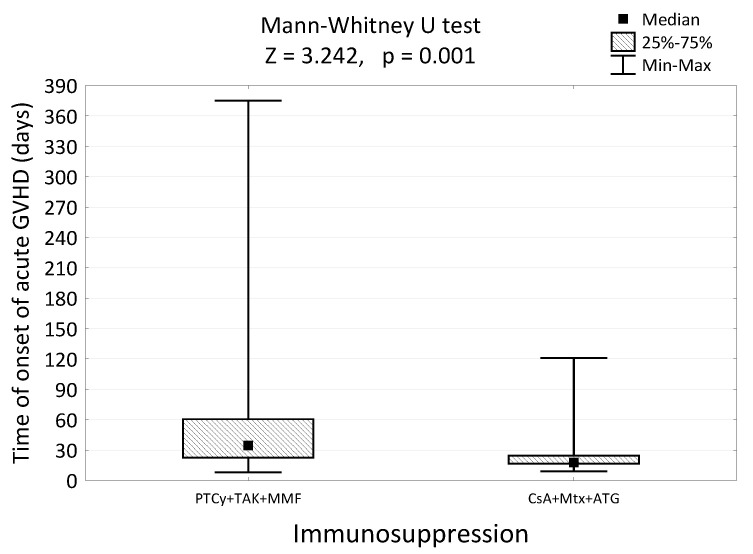
Time of onset of acute GvHD (days) in patients receiving either PTCy + TAK + MMF or CsA + MTX + ATG and the results of significance tests.

In this study, we classified 145 patients into two groups, based on the type of received immunosuppression: PTCy + TAK + MMF (110/145; 75.8%) and CsA + Mtx + ATG (35/145; 24.2%) (Table 1). Considering patients who received PTCy + TAK + MMF (110/145), 84.5% of them underwent hematopoietic stem cell transplantation from haploidentical donors (haplo-HSCT; 93/110, 84.5%), while 15.5% underwent MMUD-HSCT (17/110, 15.5%). Considering patients who received CsA + Mtx + ATG (35/145), all of them received MMUD-HSCT (35/35; 100%) (Table 1). While analyzing the MMUD group only receiving PTCy + TAK + MMF (17) vs. CsA + Mtx + ATG (35), we did not find a prevalence of any HLA locus mismatch, and, in consequence in the GvHD rate (Figure 1). The survival rate was more than eight times greater among patients receiving PTCy + TAK + MMF compared to patients receiving CsA + Mtx + ATG in the MMUD group alone (OR = 8.31 CI95% [1.97–34.9], *p* = 0.003, Table 2).

### 2.1. GvHD

**Figure 2:**
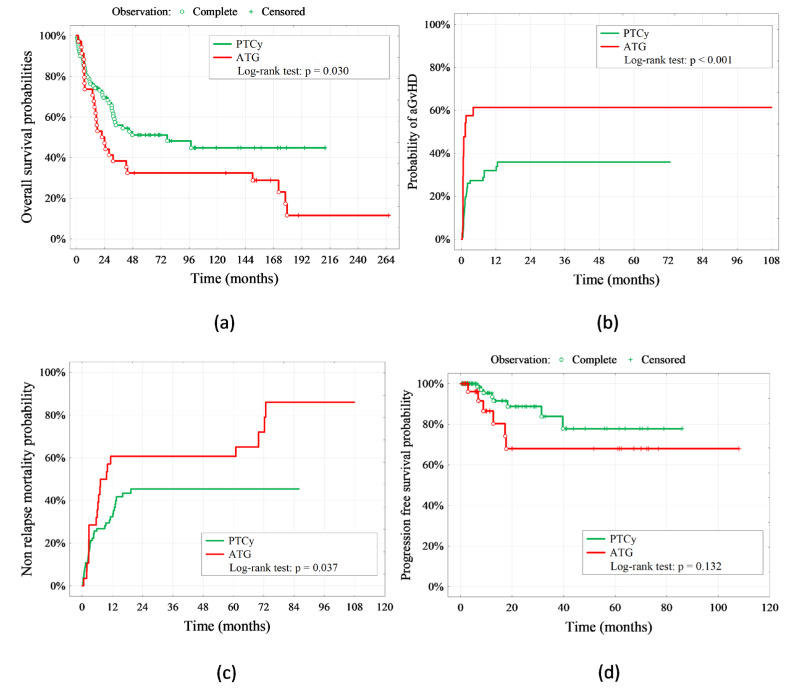
*Kaplan–Meier curves for*: (**a**) OS (overall survival), (**b**) aGvHD incidence, (**c**) NRM (nonrelapse mortality), (**d**) PFS (progression-free survival).

Of a total of 145 patients, 55 had acute GvHD (55/145, 37.9%, Table 3). This syndrome was primarily observed among patients whose donors were more than 40 years old (OR = 2.09, [1.04–4.19], *p* = 0.036, Table 3) and those who received the CsA + Mtx + ATG for GvHD prophylaxis (OR = 3.35 [1.52–7.37], *p* = 0.002, Table 3). Furthermore, including 55 patients with acute GvHD, significantly fewer received PTCy + TAK + MMF than CsA + Mtx + ATG (40/110; 36.4% vs. 21/35; 60.0%, *p* < 0.001, Table 1, Figure 2b). The cumulative incidences of grade III aGVHD were 4/110 (3.6%) for the PTCy + TAK + MMF group and 5/35 (14.3%) for the CsA + Mtx + ATG group (*p* = 0.005, Table 1). The cumulative incidences of grade IV aGVHD were 1/110 (0.9%) for the PTCy + TAK + MMF group and 2/35 (5.7%) for the CsA + Mtx + ATG group (*p* = 0.005, Table 1). In case aGvHD occurs, first symptoms in the PTCy + TAK + MMF group were observed significantly later than in those with CsA + Mtx + ATG (35 days (8–375) vs. 18 (19–121), respectively, *p* = 0.001, Figure 1, Table 1).

When considering chronic GvHD that was observed among 23 patients (23/145, 15.8%, Table 4), donor age ≥ 40 years, and haplo-HSCT administration turned out to be the most significant predictors of this syndrome (*p* = 0.016 and *p* = 0.024, respectively, Table 4). Therefore, patients older than 40 years old and those receiving haplo-HSCT were approximately three times and four times more likely to suffer from chronic GvHD, respectively (OR = 3.31 [1.32–8.31] and OR = 4.47 [1.26–15.9], Table 4).

### 2.2. CMV Reactivation

The PTCy + TAK + MMF group displayed a lower percentage of CMV reactivation (51/110; 46.4% vs. 24/35, 68.6%, *p* = 0.022, Figure 1, Table 1), and a statistically lower number of CMV copies before and after antiviral treatment (before treatment—47/110; 42.7% vs. 24/35; 68/6%, *p* = 0.008; after treatment—12/110; 10.9% vs. 10/35; 28.6%, *p* = 0.011, Figure 1, Table 1) compared to CsA + Mtx + ATG group.

### 2.3. Survival

The overall survival of patients was higher after receiving PTCy + TAK + MMF compared to CsA + Mtx + ATG (5 years OS—51.1% vs. 32.4%, respectively, *p* = 0.03, Figure 2a, Table 1). It is also worth noting that the intensity of conditioning did not affect survival. RIC was more frequent in the PTCy + TAK + MMF group but the difference was found to be not significant when analyzing both groups (*p* = 0.070, Table 1). The five-years cumulative incidence of nonrelapse mortality was 60.7% for the CsA + Mtx + ATG group vs. 45.3% for the PTCy + TAK + MMF group (*p* = 0.037, Figure 2c). There was no significant difference between groups for progression-free survival (five years PFS—68.0% vs. 77.8%, respectively, Figure 2d).

## 3. Discussion

In cases of lack of HLA-identical siblings or unrelated donors, alternative strategies rely on haploidentical donors, mismatched unrelated donors (MMUDs), and unrelated cord blood units. However, these options have been found to be more tricky, particularly by the increased incidence of graft-versus-host diseases (GvHD) and nonrelapse mortality (NRM) [21,22,23]. Strategies aiming to improve GvHD prophylaxis have already been well introduced. For many years, the standard curative therapy was the use of in vivo T-cell depleting agents such as antithymocyte globulin (ATG) or alemtuzumab, in association with a calcineurin inhibitor and methotrexate or mycophenolate mofetil [24]. However, based on the revolutionizing study at John Hopkins, more attention to post-transplant cyclophosphamide in the haplo-HSCT setting has been paid, with significant improvements in GvHD and NRM rates, and engraftment as well [9]. More recently, several studies tested the use of these immunosuppressive agents in other transplantation settings, including MMUD-HSCT [16,19,25,26]. Therefore, although PTCs are not standard agents routinely used in MMUD-HSCT, future studies should attempt to optimize protocols for MMUD-HSCT. 

The current study compared two groups classified by different immunosuppression methods including post-transplant cyclophosphamide (PTCy), in combination with tacrolimus (TAK), and mycophenolate mofetil (MMF) and cyclosporine (CsA), methotrexate (MTX), and anti-thymocyte globulin (ATG). Among patients receiving PTCy + TAK + MMF, 93 underwent haplo-HSCT (93/110; 84.5%) and 17 underwent MMUD-HSCT (17/110; 15.5%, Table 1). By analyzing these study groups, we could determine which immunosuppressive agents were more effective for different transplantation settings. Consistent with several studies [1,2,7,11], our results indicated a more favorable use of post-transplant cyclophosphamide (PTCy), in combination with tacrolimus (TAK), and mycophenolate mofetil (MMF). It is worth keeping in mind that Table 1 shows results regardless of the type of transplantation performed. However, as PTCy is commonly used in routine practice [27], the benefits of using these immunosuppressants (as observed in our study) are significant when we assume that haplo-HSCT was performed. We observed a lower percentage of CMV reactivation in the PTCy + TAK + MMF group compared to CsA + MTX + ATG (51/110; 46.4% vs. 24/35, 68.6%, *p* = 0.022, Figure 1, Table 1). Furthermore, a statistically lower copy number of CMV before and after treatment was also observed (before treatment—47/110; 42.7% vs. 24/35; 68/6%, *p* = 0.008; after treatment—12/110; 10.9% vs. 10/35; 28.6%, *p* = 0.011, Figure 1, Table 1). Although several studies showed conflicting results in this matter, some of them are consistent with our findings. For instance, Modi et al. compared immunosuppressive agents such as PTCy and thymoglobulin in the mismatched unrelated donor (MMUD) transplants among 76 patients with acute myeloid leukemia (AML) or myelodysplastic syndrome (MDS). Consistent with our result, the rate of CMV reactivation was also lower for PTCy than thyroglobulin (20% vs. 43%, *p* = 0.007) [2]. The problem of choosing the most suitable type of immunosuppression was also addressed by Camargo et al. They compared the incidence of CMV reactivation between the PTCy MMUD group (*n* = 22), PTCy haplo-HSCT group (*n* = 19), and the ATG MMUD group (*n* = 37). For all analyzed groups, the 100-day incidence of CMV was 41%, 63%, and 77% (*p* = 0.02), suggesting a higher efficacy of PTCy in CMV prevention in recipients as compared to the ATG group [28]. In contrast, among patients reported to the Center for International Blood and Marrow Transplantation Research, Goldsmith et al. analyzed the effect of graft source and PTCy on the incidence of CMV infection on transplant outcomes. CMV infection risk was significantly higher in PTCy recipients in each transplant group leading to lower overall survival and nonrelapse mortality [29]. The aggressive role of PTCy in haplo-HSCT was also observed in Al Malki et al., who monitored CMV reactivation among 119 patients undergoing haplo-HSCT for hematological diseases using PCIe. CMV reactivation was seen in 69.2% of patients at 100 days, with the median time to reactivation at 35 days [30]. It was relatively lower, though still more than half of the study group (52%) had an incidence of CMV reactivation among the 150 patients undergoing haplo-HSCT and receiving PTCy [31]. A study similar to our study concept was also performed by Massoud et al., who compared PTCy (*n* = 123) and anti-T-cell lymphocyte globulin, ATLG (*n* = 476), after a myeloablative allogeneic peripheral blood stem cell transplant. No significant differences in the incidence of CMV reactivation in these two study groups had been found (ATLG 46%, by 50%). However, they observed higher Epstein–Barr virus reactivation in the ATLG arm. Nevertheless, there was no protocol suggesting the use of specific immunosuppressive agents likely to be superior to the other ones [32]. Despite the high percentage of CMV reactivation in haplo-HSCT (<70%), its relationship with PTCy requires further consideration. Before selecting immunosuppressive agents, it is worth considering the variable rates of immune reconstitution, which are also influenced by nontransplant-related factors, such as age or patient’s health status, etc. [28]. In addition, we did not find any statistically significant differences between the groups in the proportion of non-CMV infections.

Furthermore, our study revealed a significantly lower incidence of acute GvHD in the PTCy group than in patients with ATG in (40/110; 36.4% vs. 21/35; 60,0%, *p* < 0.001, Table 1, Figure 2b). In addition, grades III and IV acute GvHD appeared less often in the PTCy group than in the ATG group (4/100; 3.6% vs. 5/35; 14.3% for grade III aGVHD and 1/110; 0.9% vs. 2/35; 5.7% for grade IV aGVHD, *p* = 0.005, Table 1). The time of onset of acute GvHD was also statistically significant, suggesting a promising use of PTCy (35 days (8–375) vs. 18 (19–121), respectively, *p* = 0.001, Table 1, Figure 2). In general, CMV reactivation and subsequent antiviral therapy often result in an increased risk of GvHD and infectious complications [33,34,35]. Therefore, we observed in our study a lower percentage of CMV reactivation and a lower incidence of acute GvHD in the PTCy group, constituting a coherent conclusion. Moreover, another aspect comes to this conclusion—the conditioning regimen. The impact of the conditioning intensity and total body irradiation on acute GvHD is still a matter of debate. Several studies have shown that MAC conditioning is a risk factor for mucositis and gastroenteritis after HSCT, which play important roles in accelerating GvHD [36,37,38]. Our study revealed no significant difference in the conditioning regimen between the two study groups (Table 1), suggesting that the observed lower incidence of aGvHD in the PTCy group is caused by the more favorable form of this immunosuppressant alone; the conditioning regimen does not influence the decrease of aGvHD rates in the PTCy group. Solomon et al. reported that class II HLA-mismatches (including HLA-DR, HLA-DQ, or HLA-DP) are associated with improved survival after haplo-HSCT utilizing PTCy [39]. Thus, our study does not describe the situation where the predominance of one locus in the MMUD group may influence the final GvHD incidence rates in patients utilizing PTCy and, hence, HLA-mismatches did not affect the observed reduction of GvHD rates. Taken together, these results confirm the crucial role of PTCy in successful transplantation.

Consistent with our findings, Modi et al., despite not finding statistical significance in grade III-IV acute GvHD at day 100 in PTCy and thymoglobulin group (12% vs. 19.6%, *p* = 0.38), observed that PTCy was associated with a lower incidence of acute GvHD compared to thyroglobulin (hazard ratio [HR] = 2.63, *p* = 0.01). However, contrary to our study, they also observed a lower incidence of chronic GvHD after receiving PTCy at one year compared to thymoglobulin (16% vs. 49%, *p* = 0.006) [2]. Furthermore, Al. Malki also found a correlation between the chronic GvHD and MMUD-HSCT PTCy group; the moderate/severe chronic GvHD rate was 3% in the entire cohort [30]. In our study, we did not find any significant correlation between chronic GvHD and analyzed groups (*p* = 0.175, Table 1). No statistical differences for chronic GvHD were also observed between groups receiving PTCy with either haplo-HSCT or MMUD-HSCT [7]. In contrast, Gaballa et al. demonstrated a lower two-year cumulative incidence of chronic GvHD in MMUD-HSCT (24% vs. 19%) compared to haplo-HSCT [11]. 

Our study revealed that the overall survival of patients was higher after receiving PTCy + TAK + MMF compared to CsA + Mtx + ATG (5 years OS—51.1% vs. 32.4%, respectively, *p* = 0.03, Figure 2a, Table 1). This finding was analyzed statistically without differentiating the type of transplantation performed. However, when taking into account only the group of patients undergoing MMUD-HSCT, the statistical significance is even higher. We have evidenced that among the MMUD-HSCT group, the survival rate was more than eight times greater among patients receiving PTCy + TAK + MMF compared to patients receiving CsA + Mtx + ATG (OR = 8.31 CI95% [1.97–34.9], *p* = 0.003, Table 2). On the other hand, nonrelapse mortality was significantly higher in the CsA + Mtx + ATG group, though for progression-free survival, we did not observe a significant difference between groups. This result confirms the benefits of receiving PTCy not only for haplo-HSCT factors analyzed regardless of the transplantation settings), which is commonly used in routine medical practice [27], but also for MMUD-HSCT. In another study, among patients undergoing MMUD-HSCT with PTCy, Shaw et al., found a one-year OS of 76% (90% CI, 67.3 to 83.3) in the entire cohort [1]. Therefore, based on results concerning different transplant-related factors, it seems reasonable to conclude that the use of PTCy has more benefits than ATG in both haplo-HSCT and MMUD-HSCT. Although both immunosuppressive regimens provide engraftment with no differences in relapse rate, nonrelapse mortality was higher in the CsA + Mtx + ATG group. Nevertheless, more studies with a larger sample size are required to confirm the conflicting results in the literature studies.

## 4. Materials and Methods

### 4.1. Study Population

The study group consisted of 145 patients (74 males and 71 females) of which 121 were aged less than 60 years old (121/145; 83.5%) (Table S1), transplanted between 2015 and 2019 in three different Polish Centres, with mobilized peripheral blood as the only source of stem cells. Most of them were diagnosed with acute myeloid leukemia and myelodysplastic syndrome (52/145; 47.3%). Other diagnoses include lymphomas and myeloma (31/145; 28.2%), acute lymphoblastic leukemia (ALL) (15/145; 13.6%), osteomyelofibrosis (OMF), chronic myeloid leukemia (CML), and others (12/145; 10.9%). Ninety-three recipients (93/145; 64,1%) underwent haplo-HSCT while the other group (52/145; 35.9%) underwent MMUD-HSCT (Table 1). PTCy was received by 110 patients (93 in haplo and 17 in the MMUD group) and 35 patients received conventional GvHD prophylaxis based on ATG, cyclosporine (CsA), and methotrexate (Mtx) in the MMUD group only (Table 2). This was a single-centered, retrospective study. The 2-year relapse rate was 18.6% (8/34) in the PTC-y group and 26.1% (6/23) in the ATG group, however, no significant difference between the groups was found (*p* = 0.14).

### 4.2. Conditioning Regimen

The proportion of patients conditioned with a myeloablative (MAC) regimen (102/145; 70.3%) was significantly higher than either reduced intensity conditioning (RIC) regimen or nonmyeloablative conditioning (NMA) regimens (22/145; 15.2% and 21/145, 14.5% respectively, both in PTCy and ATG groups (Table 1). MAC was classically defined as conditioning with TBI ≥ 5 Gy in one single dose or TBI ≥ 8 Gy in a fractioned dose or one of the following chemotherapy agents: busulfan ≥8 mg/kg b.w. (or intravenous equivalent), Treosulfan ≥ 10 mg/m^2^, melphalan ≥ 140 mg/m^2^, or Thiotepa ≥ 10 mg/kg b.w. Nonmyeloablative regimens (NMA) were mostly fludarabine based with TBI ≤ 2 Gy or TLI or lower doses of cyclophosphamide [40]. All regimens not included in the mentioned categories were considered reduced intensity (RIC). 

### 4.3. GvHD Prophylaxis 

GvHD prophylaxis in the PTCy group was built with cyclophosphamide in a single dose of 50 mg/kg b.w. given on days +3 and +4 followed by tacrolimus and mycophenolate mofetil (110/145; 75.9%). The ATG group was given either ATG-grafalon^®^ in an average total dose of 20 mg/kg b.w. or thymoglobuline^®^ in a total dose of 4.5 mg/kg b.w supported by conventional cyclosporine and methotrexate prophylaxis (35/145; 24.1%). For acute GvHD, classical Glucksberg criteria [41] with the MAGIC consortium update [42] were used, for chronic GvHD—the NIH criteria [43].

### 4.4. Statistical Methods

The distribution of the variables was assessed using descriptive statistics. The nonparametric significance test (Mann–Whitney U) was applied for qualitative variables (nominal and ordinal), the numbers (n) and structure indexes (%) were calculated, and chi-square tests of independence were used. The comorbidity index HCT-CI was calculated according to the model study [44], though we did not find any significant difference between the groups to support the main findings. Adjusted odds ratios (ORs) and 95% confidence interval (95% CI) are reported for regression analysis. The Kaplan–Meier method was used to estimate patients’ probability of survival. ROC analysis was used for evaluating the discriminatory performance of a continuous variable. All analyses were performed using the statistical software package Statistica v.13.3 (TIBCO Software Inc. Palo Alto, CA, USA). A *p*-value of < 0.05 was considered to be statistically significant.

## Figures and Tables

**Table 1:** The characteristics of patients receiving different types of immunosuppressants, including PTCy + TAK + MMF and CsA + Mtx + ATG (in red: *p* < 0.005, data were analyzed regardless of the type of transplantation performed).

	Immunosuppression				*p* Value
	PTC-y + TAK + MMF *n* = 110		CsA + Mtx + ATG *n* = 35		
**Age, *n* (%)**					0.194
<60 years	89	80.9%	32	91.4%	
≥60 years	21	19.1%	3	8.6%	
**Sex, *n* (%)**					0.975
Male	56	50.9%	18	51.4%	
Female	54	49.1%	17	48.6%	
Diagnosis *, *n* (%)					0.740
AML + MDS	52	47.3%	14	40.0%	
ALL	15	13.6%	7	20.0%	
HL + NHL + MM	31	28.2%	11	31.4%	
OMF, CML, SAA, et al.	12	10.9%	3	8.6%	
**The advancement of the disease, *n* (%)**					0.074
Remission	72	65.5%	17	48.6%	
Active	38	34.5%	18	51.4%	
CMV IgG *, *n* (%)					0.889
Negative	20	18.2%	6	17.1%	
Positive	90	81.8%	29	82.9%	
**Donor age, *n* (%)**					0.260
<40 years	67	60.9%	25	71.4%	
≥40 years	43	39.1%	10	28.6%	
**Donor sex, *n* (%)**					0.718
Male	75	68.2%	25	71.4%	
Female	35	31.8%	10	28.6%	
Conditioning *, *n* (%)					0.070
RIC	19	17.3%	2	5.7%	
MAC	72	65.5%	30	85.7%	
NMA	19	17.3%	3	8.6%	
**Unrelated donor, *n* (%)**					<0.001
Haplo	93	84.5%	0	0.0%	
MMUD	17	15.5%	35	100.0%	
**Acute GvHD, *n* (%)**					<0.001
Yes	40	36.4%	21	60.0%	
No	70	63.6%	14	40.0%	
**The degree of acute GvHD, *n* (%)**					0.005
0	76	69.1%	14	40.0%	
1	15	13.6%	10	28.6%	
2	14	12.7%	4	11.4%	
3	4	3.6%	5	14.3%	
4	1	0.9%	2	5.7%	
**Chronic GvHD, *n* (%)**					0.175
Yes	20	18.2%	3	8.6%	
No	90	81.8%	32	91.4%	
**CMV reactivation, *n* (%)**					0.022
Yes	51	46.4%	24	68.6%	
No	59	53.6%	11	31.4%	
**CMV copy before treatment, *n* (%)**					0.008
<250 copies	63	57.3%	11	31.4%	
≥250 copies	47	42.7%	24	68.6%	
**CMV copy after treatment, *n* (%)**					0.011
<250 copies	98	89.1%	25	71.4%	
≥250 copies	12	10.9%	10	28.6%	
**Current patient status, *n* (%)**					0.006
Alive	67	60.9%	12	34.3%	
Died	43	39.1%	23	65.7%	
**Median time of onset of aGvHD, (range)**	35 (8–375)		18 (9–121)		0.001
**5-years overall survival S (t = 5)**	51.1%		32.4%		0.03
**Median survival function (months)**	35		9		
**Median age, y (range)**	46 (20–70)		45 (19–71)		0.470

* AML—acute myeloid leukemia, MDS—myelodysplastic syndromes, ALL—acute lymphoblastic leukemia, HL—Hodgkin lymphoma, NHL—Non-Hodgkin’s lymphoma, MM—multiple myeloma, OMF-osteomyelofibrosis, CML—chronic myeloid leukemia, SAA—severe aplastic anemia, CMV—cytomegalovirus, RIC—reduced-intensity conditioning, MAC—myeloablative conditioning, NMA—nonmyeloablative conditioning.

**Table 2:** GvHD incidence and survival rate in MMUD patients receiving PTCy + TAK + MMF and CsA + Mtx + ATG (in red: *p* < 0.005).

Within the MMUD Group	PTC-y + TAK + MMF (*n* = 16)		CsA + Mtx + ATG (*n* = 35)		*p*-Value	OR (95% CI)
GvHD, *n* (%)					0.136	-
A	4	25.0%	12	34.3%		
B	5	31.1%	3	8.6%		
C	4	25.0%	12	34.3%		
DQ	1	6.3%	8	22.9%		
DQ + A	1	6.3%	0	0.0%		
DQ allel	1	6.3%	0	0.0%		
Patient status, *n* (%)					0.003	
Alive	13	81.2%	12	34.3%		8.31 (1.97–34.9)
Died	3	18.8%	23	65.7%		1.00 (ref.)

**Table 3:** The characteristics of patients differing by the presence of acute GvHD in a study population (in red: *p* < 0.005).

Acute GvHD Predictors	aGvHD				*p* Value
	Yes, *n* = 55		No, *n* = 90		
**Age < 60 years, *n* (%)**	48	87.3%	73	81.1%	0.460
**Female, *n* (%)**	29	52.7%	42	46.7%	0.479
Diagnosis *, *n* (%)					0.768
AML + MDS	22	40.0%	44	48.9%	
ALL	9	16.4%	13	14.4%	
HL + NHL + MM	18	32.7%	24	26.7%	
OMF, CML, SAA, et al.	6	10.9%	9	10.0%	
ELN cytogenetic risk *, *n* (%)					0.209
Favorable	0	0.0%	2	2.2%	
Intermediate	13	23.6%	17	18.9%	
Adverse	5	9.1%	18	20.0%	
Disease other than AML or MDS	37	67.3%	53	58.9%	
Negative CMV IgG *, *n* (%)	12	21.8%	14	15.6%	0.340
**Donor age ≥ 40 years, *n* (%)**	26	47.3%	27	30.0%	0.036
**Female donor**	18	32.7%	27	30.0%	0.730
HLA locus with a mismatch*, *n* (%)					0.288
A	8	14.5%	8	8.9%	
B	3	5.5%	5	5.6%	
C	10	18.2%	6	6.7%	
DQ	3	5.5%	6	6.7%	
DQ + A and allel DQ,	0	0.0%	2	2.2%	
0	31	56.4%	63	70.0%	
**Positive donor status CMV IgG, *n* (%)**	42	76.4%	60	66.7%	0.215
**Haplo**	31	56.4%	62	68.9%	0.127
**CMV reactivation, *n* (%)**	31	56.4%	44	48.9%	0.382
**≥250 copies CMV before treatment, *n* (%)**	30	54.6%	41	45.6%	0.293
**≥250 copies CMV after treatment, *n* (%)**	11	20.0%	11	12.2%	0.205
**Remission of the disease, *n* (%)**	37	67.3%	52	57.8%	0.255
**Complete remission number, *n* (%)**					0.217
4	3	5.9%	0	0.0%	
3	2	3.9%	3	3.5%	
2	10	19.6%	14	16.3%	
1	20	39.2%	36	41.9%	
**GvHD prophylaxis: CsA + Mtx + ATG**	21	38.2%	14	15.6%	0.002
Conditioning *, *n* (%)					0.543
RIC	10	18.2%	11	12.2%	
MAC	36	65.5%	66	73.3%	
NMA	9	16.4%	13	14.4%	
**Median CD34+ count, × 10^8^/kg (range)**	7.1 [4.9–9.6]		7.4 [5.4–9.9]		0.442
**The first day post-transplant when a total neutrophil count > 0.5**	17 [15–20]		19 [15–22]		0.206
**Median time between transplant and CMV reactivation, days (range)**	34 [31–48]		38 [30–49]		0.601
**<35 days between transplant and CMV, *n* (%)**	16	51.6%	13	29.6%	0.053

* AML—acute myeloid leukemia, MDS—myelodysplastic syndromes, ALL—acute lymphoblastic leukemia, HL—Hodgkin lymphoma, NHL—Non-Hodgkin’s lymphoma, MM—multiple myeloma, OMF-osteomyelofibrosis, CML—chronic myeloid leukemia, SAA—severe aplastic anemia, ELN—European LeukemiaNet, HLA—human leukocyte antigen, CMV—cytomegalovirus, RIC—reduced-intensity conditioning, MAC—myeloablative conditioning, NMA—nonmyeloablative conditioning.

**Table 4:** The characteristics of patients differing by the presence of chronic GvHD in a study population (in red: *p* < 0.005).

Chronic GvHD Predictors	cGvHD				*p* Value
	Yes *n* = 23		No *n* = = 122		
**Age <60 years, *n* (%)**	16	69.6%	105	86.1%	0.051
**Female, *n* (%)**	11	47.8%	60	49.2%	0.905
Diagnosis *, *n* (%)					0.709
AML + MDS	12	52.2%	54	44.3%	
ALL	4	17.4%	18	14.8%	
HL + NHL + MM	6	26.1%	36	29.5%	
OMF, CML, SAA, et al.	1	4.3%	14	11.5%	
ELN cytogenetic risk *, *n* (%)					0.593
Favorable	0	0.0%	2	1.6%	
Intermediate	7	30.4%	23	18.9%	
Adverse	3	13.0%	20	16.4%	
Disease other than AML or MDS	13	56.5%	77	63.1%	
Negative CMV * IgG, *n* (%)	3	13.0%	23	18.9%	0.767
**Donor age ≥ 40 years, *n* (%)**	14	60.9%	39	32.0%	0.016
**Female donor**	6	26.1%	39	32.0%	0.754
HLA * locus with a mismatch, *n* (%)					0.289
A	0	0.0%	16	13.1%	
B	1	4.3%	7	5.7%	
C	2	8.7%	14	11.5%	
DQ	0	0.0%	9	7.4%	
DQ + A and allel DQ	0	0.0%	2	1.6%	
0	20	87.0%	74	60.7%	
**Positive donor status CMV IgG, *n* (%)**	15	65.2%	87	71.3%	0.557
**Haplo**	20	87.0%	73	59.8%	0.024
**CMV reactivation, *n* (%)**	12	52.2%	63	51.6%	0.962
≥250 copies CMV before treatment, *n* (%)	11	47.8%	60	49.2%	0.905
≥250 copies CMV after treatment, *n* (%)	5	21.7%	17	13.9%	0.348
Remission of the disease, *n* (%)	17	73.9%	72	59.0%	0.178
**Complete remission number, *n* (%)**					0.065
4	2	10.0%	1	0.9%	
3	1	5.0%	4	3.4%	
2	3	15.0%	21	17.9%	
1	10	50.0%	46	39.3%	
**GvHD prophylaxis: CsA + Mtx + ATG**	3	13.0%	32	26.2%	0.276
Conditioning *, *n* (%)					0.060
RIC	7	30.4%	14	11.5%	
MAC	13	56.5%	89	73.0%	
NMA	3	13.0%	19	15.6%	
**Median CD34+ count, × 10^8^/kg (range)**	5.9 [4.9–8.8]		7.4 [5.4–9.9]		0.189
**The first day post-transplant when a total neutrophil count > 0.5**	17 [15–20]		18 [15–22]		0.959
**Median time between transplant and CMV reactivation, days (range)**	44 [30–80]		38 [30–49]		0.422
**<35 days between transplant and CMV, *n* (%)**	4	33.3%	25	39.7%	0.757

* AML—acute myeloid leukemia, MDS—myelodysplastic syndromes, ALL—acute lymphoblastic leukemia, HL—Hodgkin lymphoma, NHL—Non-Hodgkin’s lymphoma, MM—multiple myeloma, OMF-osteomyelofibrosis, CML—chronic myeloid leukemia, SAA—severe aplastic anemia, ELN—European LeukemiaNet, HLA—human leukocyte antigen, CMV—cytomegalovirus, RIC—reduced-intensity conditioning, MAC—myeloablative conditioning, NMA—nonmyeloablative conditioning.

## Data Availability

The authors confirm that the data supporting the findings of this study are available within the article.

## Supplementary Materials

The following supporting information can be downloaded at: https://www.mdpi.com/article/10.3390/ijms24065764/s1.

## References

[1] Shaw B.E., Jimenez-Jimenez A.M., Burns L.J., Logan B.R., Khimani F., Shaffer B.C., Shah N.N., Mussetter A., Tang X.Y., McCarty J.M. (2021). National Marrow Donor Program–Sponsored Multicenter, Phase II Trial of HLA-Mismatched Unrelated Donor Bone Marrow Transplantation Using Post-Transplant Cyclophosphamide. J. Clin. Oncol..

[2] Modi D., Kondrat K., Kim S., Deol A., Ayash L., Ratanatharathorn V., Uberti J.P. (2021). Post-transplant Cyclophosphamide Versus Thymoglobulin in HLA-Mismatched Unrelated Donor Transplant for Acute Myelogenous Leukemia and Myelodysplastic Syndrome. Transplant. Cell. Ther..

[3] Gragert L., Eapen M., Williams E., Freeman J., Spellman S., Baitty R., Hartzman R., Rizzo J.D., Horowitz M., Confer D. (2014). HLA Match Likelihoods for Hematopoietic Stem-Cell Grafts in the U.S. Registry. N. Engl. J. Med..

[4] Mussetti A., Kanate A.S., Wang T., He M., Hamadani M., Finel H., Boumendil A., Glass B., Castagna L., Blaise D. (2021). Haploidentical Vs. Matched Unrelated Donor Transplants Using Post-Transplant Cyclophosphamide for Lymphoma: A Joint CIBMTR/EBMT Study. Blood.

[5] Cho B.S., Min G.J., Park S., Park S.S., Shin S.H., Yahng S.A., Jeon Y.W., Yoon J.H., Lee S.E., Eom K.S. (2021). Haploidentical vs matched unrelated donor transplantation for acute myeloid leukemia in remission: A prospective comparative study. Am. J. Hematol..

[6] Passweg J.R., Baldomero H., Bader P., Bonini C., Duarte R.F., Dufour C., Gennery A., Kröger N., Kuball J., Lanza F. (2017). Use of haploidentical stem cell transplantation continues to increase: The 2015 European Society for Blood and Marrow Transplant activity survey report. Bone Marrow Transplant..

[7] Battipaglia G., Galimard J.E., Labopin M., Raiola A.M., Blaise D., Ruggeri A., Koc Y., Gülbas Z., Vitek A., Sica S. (2022). Post-transplant cyclophosphamide in one-antigen mismatched unrelated donor transplantation versus haploidentical transplantation in acute myeloid leukemia: A study from the Acute Leukemia Working Party of the EBMT. Bone Marrow Transplant..

[8] Brunstein C.G., Fuchs E.J., Carter S.L., Karanes C., Costa L.J., Wu J., Devine S.M., Wingard J.R., Aljitawi O.S., Cutler C.S. (2011). Alternative donor transplantation after reduced intensity conditioning: Results of parallel phase 2 trials using partially HLA-mismatched related bone marrow or unrelated double umbilical cord blood grafts. Blood.

[9] Luznik L., O’Donnell P.V., Symons H.J., Chen A.R., Leffell M.S., Zahurak M., Gooley T.A., Piantadosi S., Kaup M., Ambinder R.F. (2008). HLA-Haploidentical Bone Marrow Transplantation for Hematologic Malignancies Using Nonmyeloablative Conditioning and High-Dose, Posttransplantation Cyclophosphamide. Biol. Blood Marrow Transplant..

[10] Mehta R.S., Saliba R.M., Ghanem S., Alousi A.M., Rondon G., Anderlini P., Al-Atrash G., Bashir Q., Hosing C.M., Im J.S. (2022). Haploidentical versus Matched Unrelated versus Matched Sibling Donor Hematopoietic Cell Transplantation with Post-Transplantation Cyclophosphamide. Transplant. Cell. Ther..

[11] Gaballa S., Ge I., El Fakih R., Brammer J.E., Kongtim P., Tomuleasa C., Wang S.A., Lee D., Petropoulos D., Cao K. (2016). Results of a 2-arm, phase 2 clinical trial using post-transplantation cyclophosphamide for the prevention of graft-versus-host disease in haploidentical donor and mismatched unrelated donor hematopoietic stem cell transplantation. Cancer.

[12] Kröger N., Zabelina T., Binder T., Ayuk F., Bacher U., Amtsfeld G., Lellek H., Schrum J., Erttmann R., Eiermann T. (2009). HLA-Mismatched Unrelated Donors as an Alternative Graft Source for Allogeneic Stem Cell Transplantation after Antithymocyte Globulin-Containing Conditioning Regimen. Biol. Blood Marrow Transplant..

[13] Devillier R., Fürst S., Crocchiolo R., El-Cheikh J., Castagna L., Harbi S., Granata A., D’Incan E., Coso D., Chabannon C. (2014). A conditioning platform based on fludarabine, busulfan, and 2 days of rabbit antithymocyte globulin results in promising results in patients undergoing allogeneic transplantation from both matched and mismatched unrelated donor. Am. J. Hematol..

[14] Ayuk F., Diyachenko G., Zabelina T., Panse J., Wolschke C., Eiermann T., Binder T., Fehse B., Erttmann R., Kabisch H. (2008). Anti-thymocyte globulin overcomes the negative impact of HLA mismatching in transplantation from unrelated donors. Exp. Hematol..

[15] Finke J., Bethge W.A., Schmoor C., Ottinger H.D., Stelljes M., Zander A.R., Volin L., Ruutu T., Heim D.A., Schwerdtfeger R. (2009). Standard graft-versus-host disease prophylaxis with or without anti-T-cell globulin in haematopoietic cell transplantation from matched unrelated donors: A randomised, open-label, multicentre phase 3 trial. Lancet Oncol..

[16] Mielcarek M., Furlong T., O’Donnell P.V., Storer B.E., McCune J.S., Storb R., Carpenter P.A., Flowers M.E.D., Appelbaum F.R., Martin P.J. (2016). Posttransplantation cyclophosphamide for prevention of graft-versus-host disease after HLA-matched mobilized blood cell transplantation. Blood.

[17] Luznik L., Bolaños-Meade J., Zahurak M., Chen A.R., Smith B.D., Brodsky R., Huff C.A., Borrello I., Matsui W., Powell J.D. (2010). High-dose cyclophosphamide as single-agent, short-course prophylaxis of graft-versus-host disease. Blood.

[18] Kasamon Y.L., Ambinder R.F., Fuchs E.J., Zahurak M., Rosner G.L., Bolaños-Meade J., Levis M.J., Gladstone D.E., Huff C.A., Swinnen L.J. (2017). Prospective study of nonmyeloablative, HLA-mismatched unrelated BMT with high-dose posttransplantation cyclophosphamide. Blood Adv..

[19] Jorge A.S., Suárez-Lledó M., Pereira A., Gutierrez G., Fernández-Avilés F., Rosiñol L., Llobet N., Solano T., Urbano-Ispízua Á., Rovira M. (2018). Single Antigen–Mismatched Unrelated Hematopoietic Stem Cell Transplantation Using High-Dose Post-Transplantation Cyclophosphamide Is a SuiTable Alternative for Patients Lacking HLA-Matched Donors. Biol. Blood Marrow Transplant..

[20] Mehta R.S., Saliba R.M., Chen J., Rondon G., Hammerstrom A.E., Alousi A., Qazilbash M., Bashir Q., Ahmed S., Popat U. (2016). Post-transplantation cyclophosphamide versus conventional graft-versus-host disease prophylaxis in mismatched unrelated donor haematopoietic cell transplantation. Br. J. Haematol..

[21] Anasetti C., Beatty P.G., Storb R., Martin P.J., Mori M., Sanders J.E., Donnall Thomas E., Hansen J.A. (1990). Effect of HLA incompatibility on graft-versus-host disease, relapse, and survival after marrow transplantation for patients with leukemia or lymphoma. Hum. Immunol..

[22] Petersdorf E.W., Anasetti C., Martin P.J., Gooley T., Radich J., Malkki M., Woolfrey A., Smith A., Mickelson E., Hansen J.A. (2004). Limits of HLA mismatching in unrelated hematopoietic cell transplantation. Blood.

[23] Woolfrey A., Klein J.P., Haagenson M., Spellman S., Petersdorf E., Oudshoorn M., Gajewski J., Hale G.A., Horan J., Battiwalla M. (2011). HLA-C Antigen Mismatch Is Associated with Worse Outcome in Unrelated Donor Peripheral Blood Stem Cell Transplantation. Biol. Blood Marrow Transplant..

[24] Metheny L., de Lima M. (2019). Hematopoietic stem cell transplant with HLA-mismatched grafts: Impact of donor, source, conditioning, and graft versus host disease prophylaxis. Expert Rev. Hematol..

[25] Carnevale-Schianca F., Caravelli D., Gallo S. (2021). Post-transplant cyclophosphamide and tacrolimus-mycophenolate mofetil combina- tion prevents graft-versus-host disease in allo- geneic peripheral blood hematopoietic cell transplantation from HLA-matched donors. Biol Blood Marrow Transpl..

[26] Ruggeri A., Labopin M., Bacigalupo A., Afanasyev B., Cornelissen J.J., Elmaagacli A., Itälä-Remes M., Blaise D., Meijer E., Koc Y. (2018). Post-transplant cyclophosphamide for graft-versus-host disease prophylaxis in HLA matched sibling or matched unrelated donor transplant for patients with acute leukemia, on behalf of ALWP-EBMT. J. Hematol. Oncol..

[27] McCurdy S.R., Luznik L. (2019). How we perform haploidentical stem cell transplantation with posttransplant cyclophosphamide. Blood.

[28] Camargo J.F., Ebisu Y., Jimenez-Jimenez A., Natori Y., Moroz I., Morris M.I., Alencar M., Anderson A.D., Lekakis L., Beitinjaneh A. (2021). Lower Incidence of Cytomegalovirus Reactivation Following Post-Transplantation Cyclophosphamide HLA-Mismatched Unrelated Donor Transplantation. Transplant. Cell. Ther..

[29] Goldsmith S.R., Abid M.B., Auletta J.J., Bashey A., Beitinjaneh A., Castillo P., Chemaly R.F., Chen M., Ciurea S., Dandoy C.E. (2021). Posttransplant cyclophosphamide is associated with increased cytomegalovirus infection: A CIBMTR analysis. Blood.

[30] Al Malki M.M., Tsai N.C., Palmer J., Mokhtari S., Tsai W., Cao T., Ali H., Salhotra A., Arslan S., Aldoss I. (2021). Posttransplant cyclophosphamide as GVHD prophylaxis for peripheral blood stem cell HLA-mismatched unrelated donor transplant. Blood Adv..

[31] Hebert C., Watts N., Isaac S., Kukkamalla R., Jamy O., Saad A. (2017). Cytomegalovirus Reactivation after Allogeneic Hematopoietic Stem Cell Transplantation with Post-Transplant Cyclophosphamide. Biol. Blood Marrow Transplant..

[32] Massoud R., Gagelmann N., Fritzsche-Friedland U., Zeck G., Heidenreich S., Wolschke C., Ayuk F., Christopeit M., Kröger N. (2022). Comparison of immune reconstitution between anti-T-lymphocyte globulin and posttransplant cyclophosphamide as acute graft-versus-host disease prophylaxis in allogeneic myeloablative peripheral blood stem cell transplantation. Haematologica.

[33] Takenaka K., Nishida T., Asano-Mori Y., Oshima K., Ohashi K., Mori T., Kanamori H., Miyamura K., Kato C., Kobayashi N. (2015). Cytomegalovirus Reactivation after Allogeneic Hematopoietic Stem Cell Transplantation is Associated with a Reduced Risk of Relapse in Patients with Acute Myeloid Leukemia Who Survived to Day 100 after Transplantation: The Japan Society for Hematopoietic Cell Transplantation Transplantation-related Complication Working Group. Biol. Blood Marrow Transplant..

[34] Boeckh M., Nichols W.G. (2004). The impact of cytomegalovirus serostatus of donor and recipient before hematopoietic stem cell transplantation in the era of antiviral prophylaxis and preemptive therapy. Blood.

[35] Boeckh M., Nichols W.G., Papanicolaou G., Rubin R., Wingard J.R., Zaia J. (2003). Cytomegalovirus in hematopoietic stem cell transplant recipients: Current status, known challenges, and future strategies. Biol. Blood Marrow Transplant..

[36] Rapoport A.P., Miller Watelet L.F., Linder T., Eberly S., Raubertas R.F., Lipp J., Duerst R., Abboud C.N., Constine L., Andrews J. (1999). Analysis of factors that correlate with mucositis in recipients of autologous and allogeneic stem-cell transplants. J. Clin. Oncol..

[37] Hill G.R., Crawford J.M., Cooke K.R., Brinson Y.S., Pan L., Ferrara J.L.M. (1997). Total body irradiation and acute graft-versus-host disease: The role of gastrointestinal damage and inflammatory cytokines. Blood.

[38] Hill G.R., Ferrara J.L.M. (2000). The primacy of the gastrointestinal tract as a target organ of acute graft-versus-host disease: Rationale for the use of cytokine shields in allogeneic bone marrow transplantation. Blood.

[39] Solomon S.R., Aubrey M.T., Zhang X., Jackson K.C., Morris L.E., Holland H.K., Solh M.M., Bashey A. (2020). Class II HLA mismatch improves outcomes following haploidentical transplantation with posttransplant cyclophosphamide. Blood Adv..

[40] Bacigalupo A., Ballen K., Rizzo D., Giralt S., Lazarus H., Ho V., Apperley J., Slavin S., Pasquini M., Sandmaier B.M. (2009). Defining the Intensity of Conditioning Regimens: Working Definitions. Biol. Blood Marrow Transplant..

[41] Glucksberg H., Storb R., Fefer A., Buckner C.D., Neiman P.E., Clift R.A., Lerner K.G., Thomas E.D. (1974). Clinical manifestations of graft-versus-host disease in human recipients of marrow from hl-a-matched sibling donors1. Transplantation.

[42] Harris A.C., Young R., Devine S., Hogan W.J., Ayuk F., Bunworasate U., Chanswangphuwana C., Efebera Y.A., Holler E., Litzow M. (2016). International, Multicenter Standardization of Acute Graft-versus-Host Disease Clinical Data Collection: A Report from the Mount Sinai Acute GVHD International Consortium. Biol. Blood Marrow Transplant..

[43] Jagasia M.H., Greinix H.T., Arora M., Williams K.M., Wolff D., Cowen E.W., Palmer J., Weisdorf D., Treister N.S., Cheng G.S. (2015). National Institutes of Health Consensus Development Project on Criteria for Clinical Trials in Chronic Graft-versus-Host Disease: I. The 2014 Diagnosis and Staging Working Group Report. Biol. Blood Marrow Transplant..

[44] Sorror M.L., Maris M.B., Storb R., Baron F., Sandmaier B.M., Maloney D.G., Storer B. (2005). Hematopoietic cell transplantation (HCT)-specific comorbidity index: A new tool for risk assessment before allogeneic HCT. Blood.

