# Supplementary material for: The Benefits of the Post-Transplant Cyclophosphamide in Both Haploidentical and Mismatched Unrelated Donor Setting in Allogeneic Stem Cells Transplantation
Source: Int J Mol Sci. 2023 Mar 17;24(6):5764. doi: 10.3390/ijms24065764 (PMC10051342; doi:10.3390/ijms24065764)
Supplement: Supplementary file 1 [file ijms-24-05764-s001.zip › ijms-2149969-supplementary.pdf]

Table S1. Patient-, disease-, and transplantation-related characteristics

	Haplo-HSCT (n = 93)		MMUD-HSCT (n = 52)		P-value
Median age, y (range)	45 (20-70)		46 (19-71)		0.611
Age, n (%)					0.225
<60 years	75	80.6%	46	88.5%	
≥60 years	18	19.4%	6	11.5%	
Sex, n (%)					0.379
Male	50	53.8%	24	46.2%	
Female	43	46.2%	28	53.8%	
Diagnosis, n (%)					0.396
AML+MDS	42	45.2%	24	46.2%	
ALL	14	15.1%	8	15.4%	
HL+NHL+MM	30	32.3%	12	23.1%	
OMF, CML, SAA et al.	7	7.5%	8	15.4%	
The advancement of the disease, n (%)					0.035
Remission	63	67.7%	26	50.0%	
Active	30	32.3%	26	50.0%	
Complete remission number, n (%)					0.255
0	29	31.2%	20	38.5%	
1	39	41.9%	17	32.7%	
2	15	16.1%	9	17.3%	
3	5	5.4%	0	0.0%	
4	2	2.2%	1	1.9%	
N/A	3	3.2%	5	9.6%	
CMV IgG, n (%)					0.872
Negative	16	17.2%	10	19.2%	
Positive	77	82.8%	42	80.8%	
Median donor age, years (range)	35 (17-69)		32 (19-63)		0.023
Donor age, n (%)					0.031
<40 years	53	57,0%	39	75,0%	
≥40 years	40	43,0%	13	25,0%	
Donor sex, n (%)					0.959
Male	64	68,8%	36	69,2%	
Female	29	31,2%	16	30,8%	
Locus with a mismatch, n (%)					<0.001
A	0	0.0%	16	30.8%	
B	0	0.0%	8	15.4%	
C	0	0.0%	16	30.8%	
DQ	0	0.0%	9	17.3%	
DQ+A	0	0.0%	1	1.9%	
allel DQ, DP nieperm	0	0.0%	1	1.9%	
0	93	100.0%	1	1.9%	
Donor status CMV IgG , n (%)					0.034
Negative	22	23.7%	21	40.4%	
Positive	71	76.3%	31	59.6%	
GVHD prophylaxis, n (%)					<0.001

PTCy+TAK+MMF	93	100.0%	17	32.7%	
CsA+Mtx+ATG	0	0.0%	35	67.3%	
CMV prophylaxis, n (%)					0.309
Acyclovir	92	98.9%	51	98.1%	
Gancyklovir	1	1.1%	0	0.0%	
Acyclovir and letermovir	0	0.0%	1	1.9%	
Conditioning, n (%)					<0.001
RIC	19	20.4%	2	3.8%	
MAC	55	59.2%	47	90.4%	
NMA	19	20.4%	3	5.8%	
Median CD34+ count, x10^8/kg (range)	7.4 (3.3-22.8)		7.3 (3.3-14.7)		0.143
The first day post-transplant when a total neutrophil count > 0.5	19 (0-79)		16 (0-43)		<0.001
Acute GVHD, n (%)					0.127
Yes	31	33.3%	24	46.2%	
No	62	66.7%	28	53.8%	
The degree of acute GVHD, n (%)					0.053
0	62	66.7%	28	53.8%	
1 lub 2	27	29.0%	16	30.8%	
Median time of onset of acute GVHD, days (range)	36 (17-375)		18 (8-121)		<0.001
Chronic GVHD, n (%)					0.016
Yes	20	21.5%	3	5.8%	
No	73	78.5%	49	94.2%	
Treatment of acute GVHD with corticosteroids, n (%)					0.567
Yes	23	25.0%	15	29.4%	
No	69	75.0%	36	70.6%	
CMV reactivation, n (%)					0.282
Yes	45	48.4%	30	57.7%	
No	48	51.6%	22	42.3%	
Median time between transplant and CMV, days (range)	41 (13-229)		34 (8-194)		0.086
Time between transplant and CMV, n (%)	n = 45		n = 30		0.009
<35 days	12	26.7%	17	56.7%	
≥35 days	33	73.3%	13	43.3%	
Median CMV copy number between treatment, count (range)	0 (0-29000)		515 (0-180000)		0.751
CMV copy before treatment, n (%)					0.220
<250 copies	51	54.8%	23	44.2%	
≥250 copies	42	45.2%	29	55.8%	
Symptoms of CMV disease, n (%)					0.616
Yes	13	14.0%	5	9.6%	
No	80	86.0%	47	90.4%	
Disease manifestation, n (%)					0.519
No	79	84.9%	47	90.4%	
Myelosuppression	8	8.6%	1	1.9%	

Liver	3	3.2%	1	1.9%	
Lung	2	2.2%	2	3.8%	
Digestive tract	1	1.1%	1	1.9%	
Treatment, n (%)					0.304
Yes	44	47.3%	30	57.7%	
No	43	46.2%	21	40.4%	
N/A	6	6.5%	1	1.9%	
Type of treatment, n (%)					0.037
No	49	52.7%	22	42.3%	
Gancyclovir	26	28.0%	25	48.1%	
Valgancyclovir	18	19.3%	5	9.6%	
Median CMV copy number after treatment, count (range)	0 (0-114000)		0 (0-8800)		0.552
CMV copy after treatment, n (%)					0.308
<250 copies	81	87.1%	42	80.8%	
≥250 copies	12	12.9%	10	19.2%	
